# Ex vivo instability of lipids in whole blood: preanalytical recommendations for clinical lipidomics studies

**DOI:** 10.1016/j.jlr.2023.100378

**Published:** 2023-04-21

**Authors:** Qingqing Wang, Miriam Hoene, Chunxiu Hu, Louise Fritsche, Robert Ahrends, Gerhard Liebisch, Kim Ekroos, Andreas Fritsche, Andreas L. Birkenfeld, Xinyu Liu, Xinjie Zhao, Qi Li, Benzhe Su, Andreas Peter, Guowang Xu, Rainer Lehmann

**Affiliations:** 1 CAS Key Laboratory of Separation Science for Analytical Chemistry, Dalian Institute of Chemical Physics, Chinese Academy of Sciences (CAS), Dalian, China; 2 University of Chinese Academy of Sciences, Beijing, China; 3 Department for Diagnostic Laboratory Medicine, Institute for Clinical Chemistry and Pathobiochemistry, University Hospital Tübingen, Tübingen, Germany; 4 Institute for Diabetes Research and Metabolic Diseases (IDM) of the Helmholtz Zentrum München at the University of Tuebingen, Tuebingen, Germany; 5 German Center for Diabetes Research (DZD), Tübingen, Germany; 6 Department of Analytical Chemistry, University of Vienna, Vienna, Austria; 7 Institute of Clinical Chemistry and Laboratory Medicine, University Hospital Regensburg, Regensburg, Germany; 8 Lipidomics Consulting Ltd., Espoo, Finland; 9 Internal Medicine 4, University Hospital Tuebingen, Tuebingen, Germany; 10 School of Computer Science & Technology, Dalian University of Technology, Dalian, China

**Keywords:** Clinical lipidomics, blood, sample collection, preanalytical, lipid stability

## Abstract

Reliability, robustness, and interlaboratory comparability of quantitative measurements is critical for clinical lipidomics studies. Lipids’ different ex vivo stability in blood bears the risk of misinterpretation of data. Clear recommendations for the process of blood sample collection are required. We studied by UHPLC-high resolution mass spectrometry, as part of the “Preanalytics interest group” of the International Lipidomics Society, the stability of 417 lipid species in EDTA whole blood after exposure to either 4°C, 21°C, or 30°C at six different time points (0.5 h–24 h) to cover common daily routine conditions in clinical settings. In total, >800 samples were analyzed. 325 and 288 robust lipid species resisted 24 h exposure of EDTA whole blood to 21°C or 30°C, respectively. Most significant instabilities were detected for FA, LPE, and LPC. Based on our data, we recommend cooling whole blood at once and permanent. Plasma should be separated within 4 h, unless the focus is solely on robust lipids. Lists are provided to check the ex vivo (in)stability of distinct lipids and potential biomarkers of interest in whole blood. To conclude, our results contribute to the international efforts towards reliable and comparable clinical lipidomics data paving the way to the proper diagnostic application of distinct lipid patterns or lipid profiles in the future.

## Graphical abstract

**Figure:**
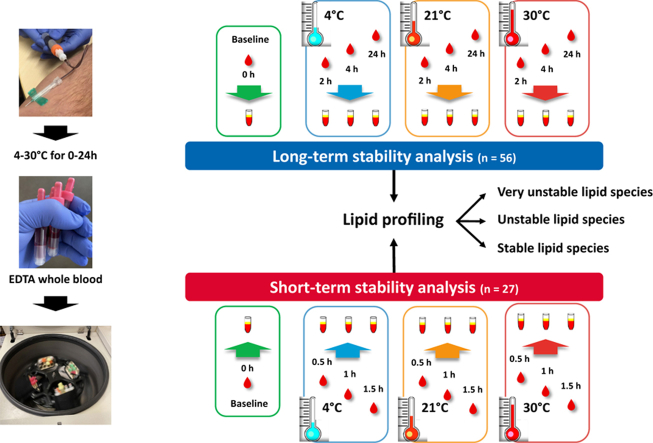

The general interest in clinical lipidomics to study lipid profiles and find new lipid biomarkers is continuously increasing, along with the collection of blood for this purpose. However, the procedures used to collect a blood sample for lipidomics analyses are not always well-defined and are pretty diverse, since preanalytical situations differ between individual hospitals or study wards. In many cases, samples are collected following standard operating procedures that are suitable for robust clinical routine parameters but not necessarily for quantifying a broad range of lipid species in blood. Not unexpectedly, discrepancies in published lipidomics data and more general issues of irreproducibility have been recognized and are debated (1, 2). Whether these differences are due to preanalytical, analytical or postanalytical issues remain unclear in most cases. To increase the interlaboratory comparability of quantitative lipid profiles, the Lipidomics Standards Initiative (LSI) and the International Lipidomics Society (ILS) aim to design guidelines for the major lipidomics workflows on a community-base, not only for the analytical and data processing/reporting phases but also for preanalytics (1, 3, 4, 5). ILS aims to pave the way for future reliable diagnostic applications of distinct lipid patterns or lipid profiles.

Effects of prolonged exposure of plasma/serum samples to adverse conditions on the stability of lipids had already been extensively studied and described (6, 7, 8, 9, 10, 11, 12, 13, 14). However, plasma/serum samples comprise the less vulnerable preanalytical phase because they are the cell-free supernatant of whole blood after centrifugation, that is, obtained at the end of the entire blood collection, handling, and transportation processes. In contrast, whole blood samples before centrifugation are a “liquid tissue” containing trillions of cells. These metabolically active cells can alter the abundance of distinct lipid species to various extents ex vivo. Consequently, handling whole blood is the most critical preanalytical step for clinical lipidomics (15). Up to now, studies investigating the stability of lipid patterns in whole blood are rare and provide only limited and incomplete information for implementation into clinical practice (7, 16, 17, 18). Uniform preanalytical recommendations about conditions and timespans for whole blood handling after collection are missing so far but would be desirable and could be one step toward, eliminating discrepancies and issues of irreproducibility in published lipidomics data.

To target preanalytical errors in lipidomics, we studied in a considerable number of samples the stability of 417 lipid species representing 13 lipid classes in EDTA whole blood at six different time points after exposure to either 4°C or 21°C or 30°C in comparison to blood samples processed at once. Three short time points (up to 1.5 h) and three longer exposures (2 h, 4 h, and 24 h) were investigated in the blood samples of 27 and 56 individuals, respectively. In addition to reporting the differences in the stability of the 417 lipid species exposed to these conditions, we discovered a potential quality control (QC) lipid triplet for the detection of sampling artifacts in the preanalytical phase from blood collection until centrifugation and separation of plasma/serum. Our data may contribute to recommendations for preanalytical guidelines for clinical lipidomics to harmonize and standardize the blood collection process.

## Materials and methods

### Standards and reagents

All method details and many other information are also provided in the attached reporting checklist of the lipidomics standards initiative (LSI) in the Supplemental Data. HPLC-grade methanol, acetonitrile (ACN), and isopropanol (IPA) were purchased from Merck (Darmstadt, Germany), HPLC-grade tert-butyl methyl ether (MTBE) and ammonium acetate from Sigma−Aldrich (St. Louis), HPLC-grade chloroform (CHCl_3_) from Duksan (Ansan-si, South Korea), and ultrapure water was prepared by a Milli-Q system (Millipore). All internal standards were purchased from Avanti Polar Lipids (Alabaster), except FA 22:0-d4 (ten Brink, Amsterdam, The Netherlands) and triacylglycerol (TG) 15:0/15:0/15:0 (Sigma-Aldrich).

### Sample collection

**Fig. 1:**
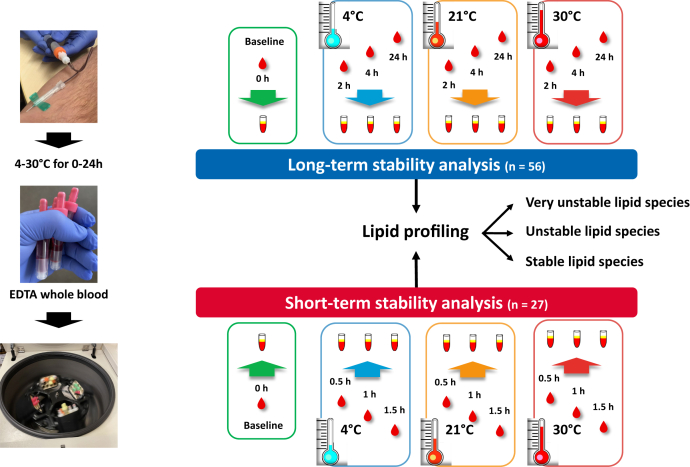
Illustration of the study design. “Short term” means exposure of whole blood from 27 individuals for 0.5 h, 1 h, and 1.5 h to either 4°C, 21°C, or 30°C. “Long term” means exposure of whole blood from 56 individuals for 2 h, 4 h, and 24 h to either 4°C, 21°C, or 30°C.

For this preanalytical study, 10 ml of EDTA whole blood were drawn from 83 randomly selected subjects by a longstanding, experienced team at the ward for metabolic studies at the University Hospital of Tuebingen. Exclusion criteria were manifest diabetes, kidney dysfunction (GFR <50 ml/min), liver disease, and systemic infection. After the drawing of blood, samples were immediately divided within 5 min into aliquots according to the scheme shown in Fig. 1. Centrifugation to separate EDTA plasma from blood cells was either performed immediately or after 0.5 h, 1 h, 1.5 h (short-term stability analysis, n = 27 subjects) or after 2 h, 4 h, or 24 h (long-term stability analysis, n = 56 subjects) at 4°C (cooled at once) or 21°C (room temperature) or 30°C (summer time conditions). At the end of the respective exposure time, whole blood was centrifuged at 4°C (3,100 *g* for 7 min), and EDTA plasma was stored at once at −80°C in 100 μl aliquots until further use. In total, 829 samples were studied (one missing sample at 2 h; 30°C). Informed written consent was obtained from all participants, and the ethics committee of the university of Tuebingen approved the protocol (ref. 247/2017BO1) according to the Declaration of Helsinki of 1964 and its later amendments.

### Sample preparation

Plasma samples were extracted using MTBE/methanol/water as described previously (19). Fifty microliters of plasma were mixed with 300 μl of MeOH containing internal standards (per 300 μl: 1,250 ng phosphatidylcholine [PC] 15:0/15:0, 500 ng lysophosphatidylcholine [LPC] 19:0, 100 ng LPC 15:0, 100 ng phosphatidylethanolamine [PE] 15:0/15:0, 25 ng phosphatidylglycerol [PG] 15:0/15:0, 500 ng SM d18:1/12:0, 100 ng ceramide [Cer] d18:1/17:0, 100 ng diacylglycerol [DG] 15:0/18:1-d7, 1 μg TG 15:0/15:0/15:0, and 250 ng FA 22:0-d4). After vortexing for 30 s, 1 ml of MTBE was added, and the mixture was vortexed for 30 min at room temperature. Subsequently, 250 μl of water was added, followed by vortexing for 30 s and incubation at 4°C for 10 min to form a two-phase system. Then, the mixture was centrifuged at 5,000 *g* at 4°C for 10 min. Two 350 μl aliquots of supernatant were evaporated to dryness and stored at −80°C. The extracted lipids were reconstituted in 30 μl of CHCl_3_/methanol (2:1, v/v), followed by dilution with ACN/IPA/water (65:30:5, v/v/v) containing 5 mM ammonium acetate. QC samples were prepared by pooling equal amounts of lipid extracts from each sample and were analyzed after every tenth sample in a sequence.

### Nontargeted UHPLC-high resolution mass spectrometry-based lipidomics

All chromatographic and MS details and many other information are also provided in the attached reporting checklist of the LSI. A UHPLC-Q Exactive MS system (Thermo Fisher Scientific, Rockford) was operated in both positive and negative ion modes using a 2.1 × 100 mm ACQUITY™ 1.7 μm BEH C8 column (Waters, Ireland). The mobile phases were ACN/water (60:40, v/v) and IPA/ACN (90/10, v/v) both with 10 mM ammonium acetate. The gradient elution started with 50% B, maintained for 1.5 min, then increased linearly to 85% B at 9 min, reached 100% B at 9.1 min, and was held for 1.9 min, finally back to 50% B within 0.1 min, and equilibrated for 1.9 min. The flow rate was 0.3 ml/min. The column temperature was maintained at 60°C. The mass spectrometer was operated with scan range m/z 300–1,100 and m/z 120–1,600 in positive and negative ion mode, respectively. The resolutions of 140,000 and 70,000 were set for full scan MS and data-dependent MS/MS in both modes. The TopN (N, the number of top most abundant ions for fragmentation) was set to 10. The normalized collision energy was set to 15, 30, and 45 eV. The MS1 automatic gain control target value was 3 × 10^6^, and the maximum injection time was 100 ms. For MS/MS scans, the automatic gain control target value was 1 × 10^5^, and the maximum injection time was 50 ms. Heated electrospray ionization source parameters were set as follows: spray voltage 3.5/-3 kV, capillary temperature 320°C, sheath gas flow rate 45 a.u. (arbitrary units), aux gas flow rate 10 a.u., S-lens RF level 50, and aux gas heater temperature 350°C. The analytical performance of this approach is illustrated in supplemental Fig. S1.

### Data preprocessing

Lipid identification was achieved according to our prior study (20), meaning MS/MS fragment, exact m/z, and retention time in combination with LipidSearch software (Thermo Fisher Scientific, Waltham). Peak areas of the detected lipids were obtained using Thermo TraceFinder and normalized by corresponding internal standards. Applying the modified 80% rule (21), the peaks showing more than 20% missing values among each group were excluded. Coefficients of variation were calculated for all peaks in QC samples to evaluate the quality of the data. Lipids with a coefficient of variation >30% were excluded from subsequent analysis.

### Statistical analysis

All statistical studies and visualization were conducted using R software (v4.1.0) unless otherwise noted. Wilcoxon matched-pairs signed rank tests were performed with MATLAB (R2014a, MathWorks, Natick, MA). False discovery rate for multiple testing was calculated with the Benjamini-Hochberg method. Significant alteration of a lipid species level was defined as >10% change in signal intensity (*P* < 0.05; FDR <0.05) in comparison to fresh, that is, immediately processed blood samples. R was also used for heat map visualization and GraphPad Prism to generate box plots. For heat map generation, missing values were imputed with one-tenth of the minimal value for every variable. Then the fold changes of different time points at different temperatures compared to fresh, that is, immediately processed samples (time point 0 h), were calculated, and log-transformed fold-change values were visualized. The strategy applied for the detection of possible sample QC biomarkers is illustrated in supplemental Fig. S2. As a first step, in parallel, two different identification approaches were performed to detect unstable lipid species. The results of these two approaches were compared and lipid species detected in both approaches were used for the next evaluation step (supplemental Fig. S2). One approach was a mixed effects linear regression analysis performed for each lipid, and the other approach was a supervised classification comparing samples processed at once (good quality) and samples originating from blood exposed for 24 h to 30°C (questionable quality) (supplemental Fig. S2). For the mixed effects linear regression analysis, log-transformed fold-change values of lipid levels at different time points and temperatures compared to immediately processed samples (time point 0 h) were used as dependent variables. The variables time point, temperature, and their interaction term were used as fixed effects. Due to three coefficients per model and 417 models, the statistical significance was set at the *P*-values of the regression coefficients with a Bonferroni corrected *P* < 3.96∗10^−5^ (*P* < 0.05/ (3∗417)). The results of mixed effects linear regression analysis and supervised classification were compared, and lipid species detected in both approaches were used for the next evaluation step by least absolute shrinkage and selection operator (LASSO) regression (22). Resulting potential QC biomarkers from LASSO regression analysis were evaluated further in a last step by backward stepwise logistic model for extensive sample classification based on a nonstringent quality definition (supplemental Fig. S2). We classified all samples generated under conditions leading to no significant changes in the total covered pattern of 417 lipids as samples of good quality (n = 220). Samples from all other collection conditions were entitled as samples of questionable quality (n = 526).

## Results

The stability of the lipidome in EDTA whole blood, that is, possible alterations during the timespan from blood drawing until centrifugation and separation of plasma, was investigated in a profile of 417 lipid species from 13 classes in a semiquantitative manner at six exposure times from 0.5 to 24 h under three different temperature conditions (4°C, 21°C, and, to reflect summer time, 30°C; Fig. 1). The time points and exposure conditions were chosen based on our practical experiences in everyday clinical situations for collection, handling, and transportation of whole blood either for diagnostic or research purposes.

**Fig 2:**
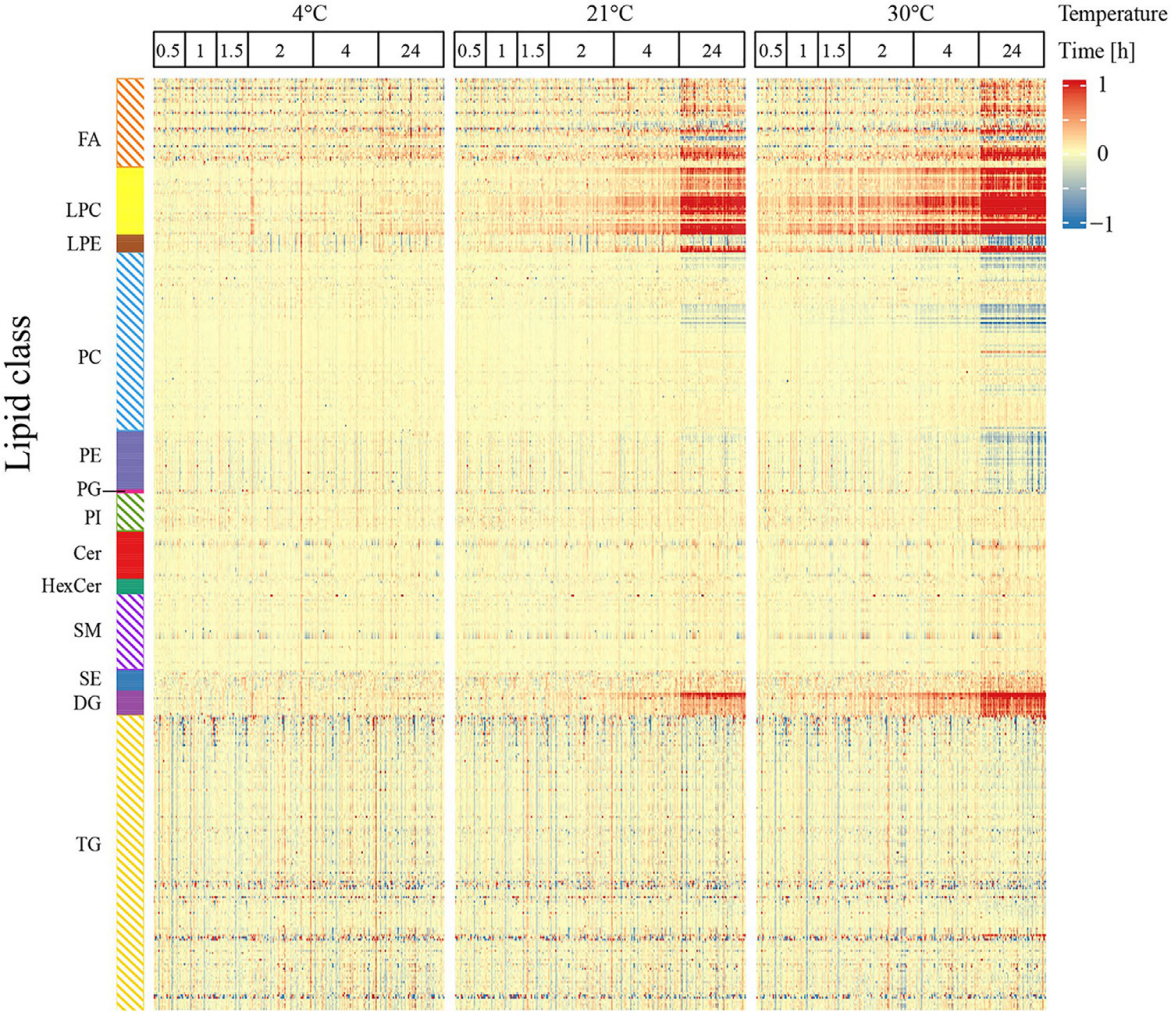
Heat map showing the time-dependent (in)stability of all 417 covered lipids in EDTA whole blood exposed to different temperatures. Six exposure times from 0.5 h to 24 h and three different temperatures (4°C, 21°C, and 30°C) were investigated, based on typical conditions during the timespan from blood drawing until centrifugation and separation of plasma in hospitals or study wards.

We detected a vast range of stabilities, reaching from very stable (24 h at 30°C) to very unstable lipids (<0.5 h at nonchilled conditions). Table 1 shows the stability of each lipid class at a glance, and Fig. 2 illustrates these findings by a heat map. A complete list of all covered lipid species is provided in supplemental Table S1.

**Table 1:** Summary of the stability of 417 lipids from 13 different classes in whole blood after collection

	Cooled at once 4°C Time [h]						Room temp. 21°C Time [h]						Summer time conditions 30°C Time [h]					
	0.5	1	1.5	2	4	24	0.5	1	1.5	2	4	24	0.5	1	1.5	2	4	24
Lipid class																		
FA (n = 40^a^)						10		1	2	2	10	25		2	4	5	14	29
LPC (n = 30)						5		5	11	12	22	27	12	19	23	25	27	29
LPE (n = 8)								1	1		3	6	1	3	3	3	5	7
PC (n = 80)												9					2	21
PE (n = 26)																		13
PG (n = 2)																		1
PI (n = 17)																		
Cer (n = 21)																		2
HexCer (n = 7)																		
SM (n = 34)																		
SE (n = 9)												6					1	7
DG (n = 11)						1				1	3	11		2	3	6	11	11
TG (n = 132)						3					3	8	1				1	9
**Sum (%)**						**19**		**7**	**14**	**15**	**41**	**92**	**14**	**26**	**33**	**39**	**61**	**129**
						**(4.6%)**		**(1.7%)**	**(3.4%)**	**(3.6%)**	**(9.8%)**	**(22.1%)**	**(3.4%)**	**(6.2%)**	**(7.9%)**	**(9.4%)**	**(14.6%)**	**(30.9%)**

The numbers in each column correspond to lipids defined as unstable according to the following criteria: *P* < 0.05 and FDR <0.05 and >10% change in signal intensities in comparison to samples prepared at once (0.5–1.5 h, n = 27 per time point; 2–24 h, n = 56 per time point). Details of each significantly altered lipid species after 24 h exposure (e.g. significance level, percentage of alteration) are provided in supplementals Table S2 and S3.

Cer, ceramide; DG, diacylglycerol, FA, free fatty acid; HexCer, hexosylceramide; LPC, lysophosphatidylcholine; LPE, lysophosphatidylethanolamine; PC, phosphatidylcholine; PE, phosphatidylethanolamine; PG, phosphatidylglycerol; PI, phosphatidylinositol; SE, cholesterol ester; SM, sphingomyelin; TG, triacylglycerol.

a total number of covered lipid species in the lipid class.

Our first interest was identifying lipids that are very stable in whole blood. No alterations at all conditions studied were detected for three classes (Table 1 and supplemental Table S1), phosphatidylinositol (17 species covered), hexosylceramide (HexCer, 7 species covered), and SM (34 species covered). Furthermore, 123 of 132 covered TG were stable. In total, 288 lipid species did not display any changes in their levels at the most challenging condition (24 h exposure of EDTA whole blood to 30°C). At room temperature, six out of 13 lipid classes (PE, PG, phosphatidylinositol, Cer, HexCer, SM) and 325 lipid species levels were unaffected after 24 h, including 124 out of 132 TG (note: only two PG species were covered).

Our subsequent interest was to find the best timespan and condition for whole blood handling and transportation, aiming to preserve the level of all covered lipids. At 4°C, no significant change of any covered lipid was detected for up to 4 h, and even in blood chilled for 24 h before plasma separation, only 19 out of the 417 lipids were altered in their levels (Table 1 and Supplemental Table S2).

**Fig. 3:**
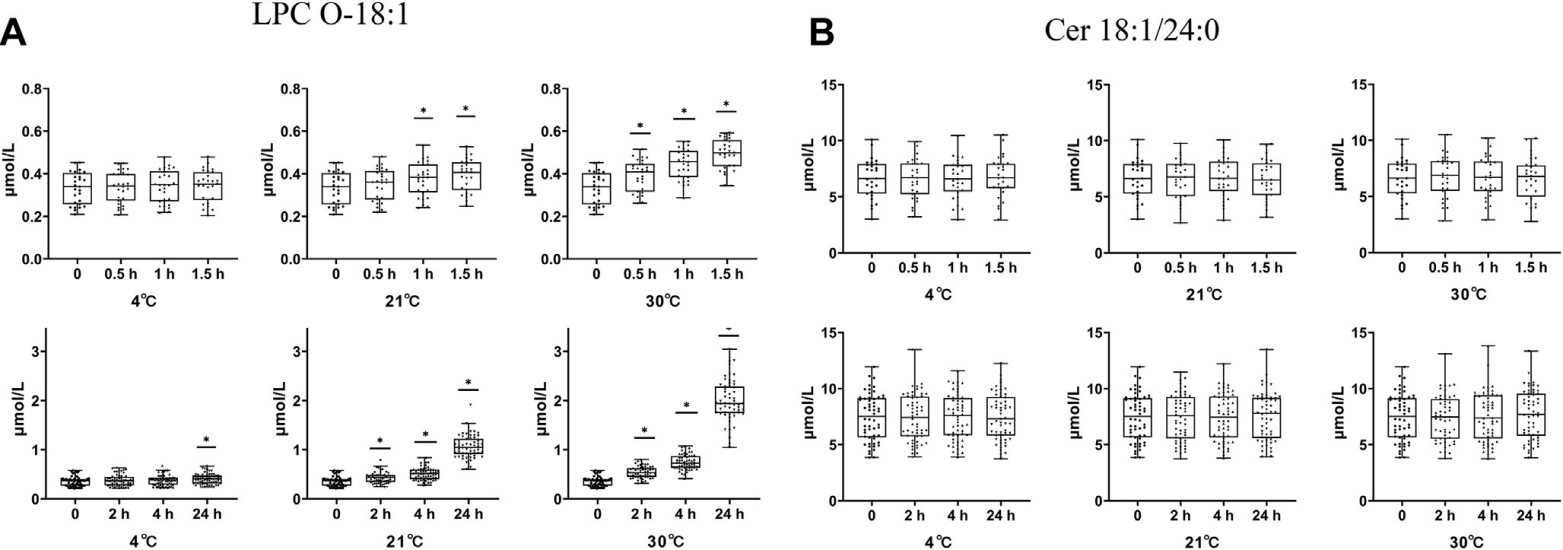
Representative examples illustrate pronounced differences in the stability of distinct lipid species in whole blood. (A) LPC O-18:1 is an unstable and (B) Cer 18:1/24:0 is a very stable lipid species. Time points up to 1.5 h, n = 27 individuals; time points 2 h, 4 h, and 24 h, n = 56 subjects (at time point 2 h (30°C), one sample is missing). LPC 19:0 for LPC O-18:1 and Cer d18:1/17:0 for Cer 18:1/24:0 from the internal standards (IS) mixture were used for normalization. ∗*P* < 0.05. Cer, ceramide; LPC, lysophosphatidylcholine.

Next, we focused on expected less stable lipids, that is, the question of which lipids are vulnerable ex vivo in whole blood during the preanalytical phase. Very rapid, after 0.5 h at 30°C, 14 out of 417 lipids changed, most strongly 12 LPC species (Table 1 and supplemental Table S3). After 24 h at 30°C, one-third of all lipid levels were altered (48 decreased, 81 increased; Table 1 and supplemental Table S2). Particularly pronounced alterations (>3-fold increases) were detected for 14 lipids, most of which were LPC (supplemental Table S2). Decreases were dominated by PC (20 out of 80), PE (13 out of 26), and TG (7 out of 132). Exposure of blood at room temperature for 4 h, a situation not unusual in hospitals, led to changes of 41 lipid species in whole blood from the classes of FA (10 out of 40), LPC (22 out of 30), lysophosphatidylethanolamine (LPE, 3 out of 8), DG (3 out of 11), and TG (3 out of 132). Figure 3 shows representative examples of an unstable and a very stable species and illustrates the pronounced differences in the stability of distinct lipids. LPC, frequently reported as biomarkers in diagnostic patterns, show very rapid and pronounced changes, as demonstrated by LPC O-18:1 (Fig. 3A). For LPC O-18:1, a more than 5.6-fold increase after 24 h exposure to 30°C was detected (supplemental Table S2). Noteworthy, this increase can be prevented for up to 4 h if the whole blood is directly cooled after drawing (Fig. 3A). Even after 24 h at 4°C, the increases in LPC O-18:1 level amounted to only 13.4% (supplemental Table S2). In contrast to LPC, the box plots of Cer d18:1/24:0 in Fig. 3B underline the preanalytical robustness of other lipids in whole blood under all studied conditions. Cer d18:1/24:0 is one of the four ceramides described as a novel diagnostic tool in cardiology (23, 24).

**Fig. 4:**
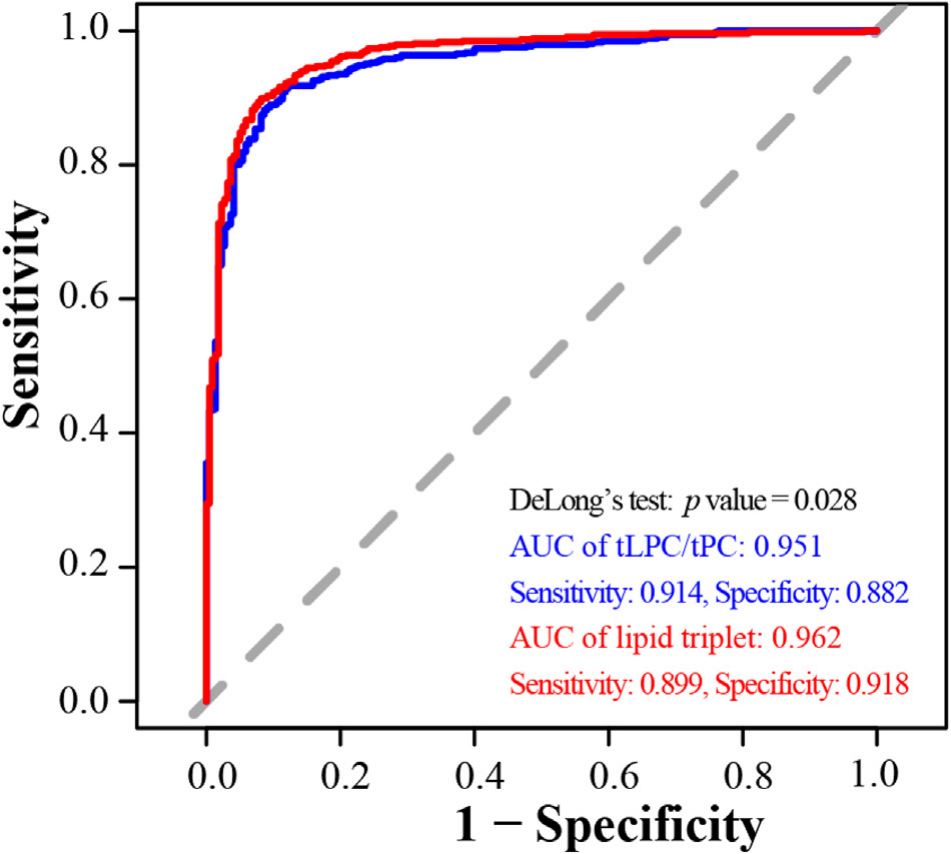
Comparison of the classification performance to assess the sample and/or data quality in clinical lipidomics studies by a lipid triplet (LPC 16:1, LPC O-18:1, and LPE 20:4) as a possible quality control biomarker pattern and an already published lipid-based QC marker, the ratio at lipid class level of LPC/PC. The classification performance is shown by an area under the receiver operating characteristic (ROC) curve (AUC) analysis. LPC, lysophosphatidylcholine; PC, phosphatidylcholine; QC, quality control.

Next, we aimed to identify a lipid pattern allowing to detect samples of questionable quality (or lipidomics data obtained from such samples) caused by shortcomings during blood collection and transportation. The stepwise-applied detection strategy is illustrated in supplemental Fig. S2, and a detailed description is given in the method section. As a first step, in parallel, two different identification approaches were performed to detect unstable lipid species, namely a mixed effects linear regression analysis and a supervised classification (supplemental Fig. S2). The results of these two approaches were compared and lipid species detected in both approaches were used for the next evaluation step (supplemental Fig. S2). In the first step, the linear regression model based on significant *P*-values related to time, temperature, or their interaction detected 149 lipids considered to be sensitive to preanalytical issues, and the other approach, the supervised classification comparing samples processed at once (good quality) and samples originating from blood exposed for 24 h to 30°C (questionable quality), led to the detection of 129 unstable lipids. The comparison of these 149 and 129 lipid species revealed 114 identical lipid species detected in both approaches, which were used for the next step, LASSO regression analysis for variable selection out of these 114 lipids (supplemental Fig. S2). LASSO regression revealed 6 lipids (LPC 16:1, LPC 17:0, LPC 18:3, LPC 20:1, LPC O-18:1, and LPE 20:4) as potential QC candidate lipid species. In the last step, a backward stepwise logistic model for extensive sample classification was applied for further variable selection (supplemental Fig. S2), finally identifying a lipid triplet, namely LPC 16:1, LPC O-18:1, and LPE 20:4, as a potential QC pattern for lipidomics studies, possibly allowing to assess the occurrence of sampling artifacts during blood collection. In a first evaluation step, the performance by an average area under the receiver operating characteristic-curve (AUC) analysis was studied. For this evaluation, all samples generated under conditions leading to no significant changes in the total covered pattern of 417 lipids were classified as samples of good (n = 220) and the others as of questionable quality (n = 526). The lipid triplet achieved an AUC of 0.959 based on a 5-fold cross-validation (10 repeats) (supplemental Fig. S3). Next, we compared the performance of the lipid triplet with one of the few lipid-based, published sample QC tools, the ratio at lipid class level of LPC/PC (14). Figure 4 shows that very similar AUCs were achieved (0.962 for the lipid triplet and 0.951 for the ratio) for the detection of the 526 samples having a quality less suitable to achieve valid lipidomics data.

## Discussion

Reliability, robustness, and interlaboratory comparability of quantitative measurements is critical for lipidomics analyses, primarily if the application of distinct lipids or patterns in blood and other body fluids is intended for clinical diagnostics. Currently, international efforts by the ILS to collect data for the generation of guidelines are on the way, aiming to harmonize and standardize all steps of the lipidomics workflow. A minimal reporting checklist has already been published (25). From our data, achieved as part of the “Preanalytics interest group” in a considerable number of samples, recommendations can be derived for the handling and transportation of whole blood. In addition, the comprehensive results in supplemental Table S2 can be of practical use to check the instability of distinct lipids including potential biomarkers of interest. Furthermore, we identified a triplet of lipids (LPC 16:1, LPC O-18:1, and LPE 20:4) potentially suitable to detect whole blood sampling artifacts, which is covered by most lipidomics approaches.

While processing plasma/serum for clinical lipidomics is generally well standardized and tightly controlled, this is not the case for blood collection. While processing plasma/serum for clinical lipidomics is generally well standardized and tightly controlled, this is not the case for blood collection. Whole blood samples before centrifugation should more or less be seen as “liquid tissue” in a collection tube containing trillions of metabolic active cells as well as extracellular enzymes (like phospholipases), free radicals, etc., altering ex vivo levels of distinct lipids in blood to various extent, since trafficking, formation, and metabolism of lipids is performed by various transporters and enzymes like lipid synthetases, ligases, phospholipases, oxidases, as well as reductases. Furthermore, unsaturated lipids can undergo facile oxidation ex vivo. Attention should be payed to the fact that particularly at room temperature, the metabolic activity of blood cells is high, as well as the activity of plasma enzymes, like for instance, lecithin-cholesterolacyltransferase, phospholipase A_1_ and A_2_ generating lysophospholipids and LPA as well as FA from phospholipids or phospholipase D cleaving glycerophospholipids into phosphatidic acids, which are converted to DGs via phosphatidic acid phosphohydrolase (26). In our study, we provide clear evidence that cooling at once after blood drawing stabilized all covered 417 lipids for up to 4 h. Even after 24 h, the levels of 398 lipids were stable in at once cooled blood. Thus, we recommend to cool blood at once and persistently until separation of plasma/serum, which should ideally be completed within 4 h, except in cases where the analytical focus is exclusively on very stable lipid species. Our recommendation is in good agreement with previous publications, which, however, provided only incomplete information for clinical lipidomics (7, 16, 17, 18, 27). A rapid increase of LPA, S1P, and SA1P in blood stored at room temperature within 1 h and its prevention by cooling was described (7). At 4°C, selected PUFA were stable in EDTA whole blood samples up to the longest studied time point of 2 h (17). Hahnefeld and coauthors showed that most of the lipids covered in their profile were stable for up to 4 h at 4°C in blood, except the endocannabinoids (N-acetylethanolamides and monoacylglycerols), which were only stable for 20 min (18). They recommended at once cooled whole blood to be processed for the measurement of endocannabinoids within 1 h and for LPA, sphingolipids, and nontargeted profiling within 2 h (18). Kamlage *et al.*(16) recommended processing blood samples as quickly as possible based on data from metabolite profiles, including lipids. Thus, for both lipidomics and metabolomics studies, cooling at once and processing within 4 h is a consistent recommendation (15, 27).

Although cooling at once and timely transportation of whole blood can be stipulated in guidelines, standard operating procedures, and protocols, there is no guarantee of its permanent fulfillment during a daily routine in hospitals. Hence, a reliable diagnostic tool should ideally contain only very robust lipid(s) to avoid that the period until centrifugation of whole blood is a critical issue when it is prolonged. We detected 325 and 288 robust lipid species in EDTA whole blood samples showing for 24 h at 21°C or even at 30°C no changes, respectively (supplemental Tables S1 and S2). Such lipids guarantee valid results even when samples are transported in an extended hospital area or by mail within 24 h. Useful (in)stability data for many published and potential future diagnostic lipid biomarkers are provided in supplemental Tables S1, S2 and S3, including, for example, a quartet of ceramides on the verge of being used in cardiologic diagnostics (23, 28, 29). Two of these ceramides are stable for 24 h at 30°C (Cer d18:1/24:0 and Cer d18:1/24:1) and the other two for 24 h at 21°C (Cer d18:1/16:0, Cer d18:1/18:0), hence common preanalytical issues will not compromise the achievement of valid results with this lipid quartet, at least in internal clinical use. However, among the numerous novel plasma/serum lipid biomarkers published so far for possible diagnostic use (30, 31, 32) are also lipids less suited from the preanalytical point of view. One recent example is a pattern of 7 lipids, including LPC 20:0, identified by Alshery *et al.* (33), which improves the prediction of cardiovascular events in type 2 diabetes. Based on our data, LPC 20:0 shows a continuous, temperature-dependent increase in whole blood (e.g. > 2.5-fold after 24 h at 30°C; supplemental Table S2), which may lead to false-high concentrations and consequently misinterpretations if preanalytical issues occur. To achieve valid results with those less stable lipids requires greater preanalytical efforts and needs, more precisely, to cool blood samples at once and process them within 4 h. This is recommended for unstable diagnostic lipid biomarkers and blood collected for nontargeted lipidomics projects. Based on our findings, FA and LPC should be interpreted with caution in all circumstances, except the preanalytical process was tightly controlled and followed the strictest recommendations of immediate cooling and processing.

The risk of misinterpretation, particularly for the less stable lipids, can be reduced or minimized by applying a QC marker to detect and exclude samples or results of questionable quality. Only very few studies have identified promising QC biomarkers reflecting deviations in sample processing (14, 16, 34, 35, 36). However, their applicability in lipidomics approaches is limited to some extent because they are either difficult to analyze, a complex pattern is suggested (14, 16), the marker is not covered in typical analysis (36), or it is not at all included in lipid profiles (34, 35). We identified a possibly suited, simple lipid triplet, consisting of LPC 16:1, LPC O-18:1, and LPE 20:4, included in most data sets of standard lipidomics procedures. The receiver operating characteristic-curve AUC performance of this possible QC tool to identify sampling artifacts is comparable to a published lipid-based QC marker (the ratio at lipid class level of LPC/PC (14)), but its application is much easier. Applying this potential QC triplet for identifying samples containing lipids altered by delays during whole blood processing or interruption of the cold chain could be feasible, but further extensive validation in other clinical and already stored biobank samples is needed (e.g. robustness, definition of cut-off levels, exclusion of effects of diseases, etc.). In particular, samples of all kinds of diseases with elevated lysospecies, like inflammatory or inflammatory-associated diseases, need to be included in these future studies to exclude marking a sample as poor quality when in fact the cause is a disease state. Regular application of sample QC markers may also contribute to increased comparability and reproducibility of clinical lipidomics data (3).

Some limitations of our study merit consideration are as follows: a) valid conclusions for a transport by mail exceeding 24 h are not possible, b) stability statements are valid for the 417 covered lipids but not necessarily for other lipids of the same class, c) deviations from our findings in the stability of some lipid species in blood of patients with severe diseases (e.g. acidotic blood) cannot be excluded, d) final conclusions about the practicability of the suggested QC triplet necessitate previous extensive validation in other clinical and biobank samples, and e) for analytical reasons, no recommendation for very unstable lipid classes (e.g., oxylipins, eicosanoids, endocannabinoids, etc.) can be provided. Furthermore, if, in contrast to our approach, no high resolution mass spectrometry is applied, the interpretation in samples of inferior quality can be more challenging because of overlapping isomeric and isobaric species, particularly in direct infusion lipidomics analyses.

To conclude, reproducible and quantitatively concordant data requires different layers of quality assurance and QC measures, as stated by the “LSI” in their position paper (3). Aiming to fulfill these requirements for the phase of whole blood handling, we recommend to cool blood samples at once and persistently and to complete centrifugation and plasma separation within 4 h. This allows to achieve valid and reproducible profiles for, at least, all lipids covered in our approach. Furthermore, QC, that is, searching for sampling artifacts during whole blood collection, should regularly be performed using QC markers. We provide information about 325 and 288 lipids with a very high ex vivo robustness in blood at room and higher temperatures, respectively. Lipids from this list could be particularly well suited for diagnostic purposes since sample treatment can be performed as for most of the other patient samples in the hospital with no need for cooling or timely processing. Overall, our findings, generated within the LSI “Preanalytics interest group,” may contribute to the international efforts to reach reliable and comparable clinical lipidomics data in the near future, independent from the place where they were generated.

## Data availability

The data sets that support the findings of this study are available in this article or the supplemental data.

## Supplemental data

This article contains supplemental data.

## Conflict of interest

The authors declare that they have no conflicts of interest with the contents of this article.
