# Supplementary material for: Ex vivo instability of lipids in whole blood: preanalytical recommendations for clinical lipidomics studies
Source: J Lipid Res. 2023 Apr 21;64(6):100378. doi: 10.1016/j.jlr.2023.100378 (PMC10208886; doi:10.1016/j.jlr.2023.100378)

Figure S1

Box-plots showing the distribution of the coefficients of variation of 417 lipids in QC samples from the data for the sample sets of short-term (A) and long-term (B) analysis, respectively . “Short term” means exposure of whole blood from 27 individuals for 0.5h, 1h and 1.5 h to either 4°C, or 21°C or 30°C. “Long term” means exposure of whole blood from 56 individuals for 2h, 4h and 24h to either 4°C, or 21°C or 30°C.

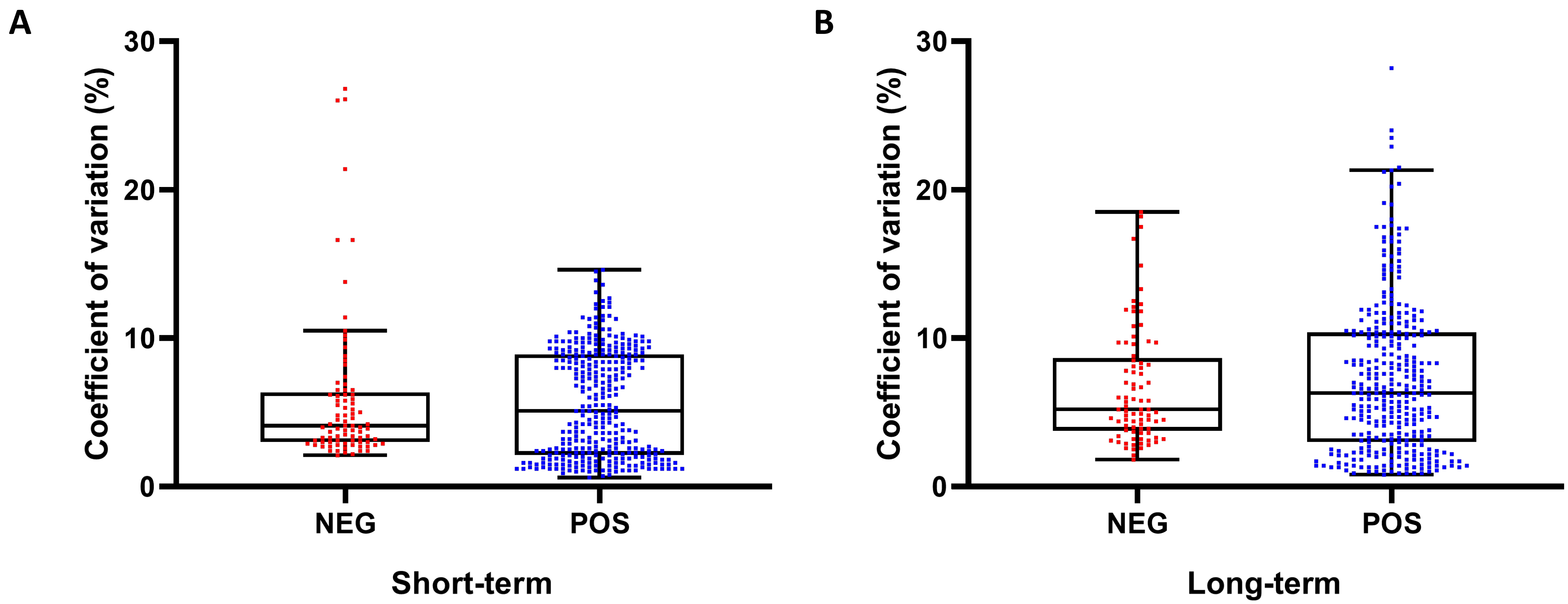

Figure S2.
Scheme of the workflow for the identification of a lipid pattern as a possible tool to assess the sample quality.

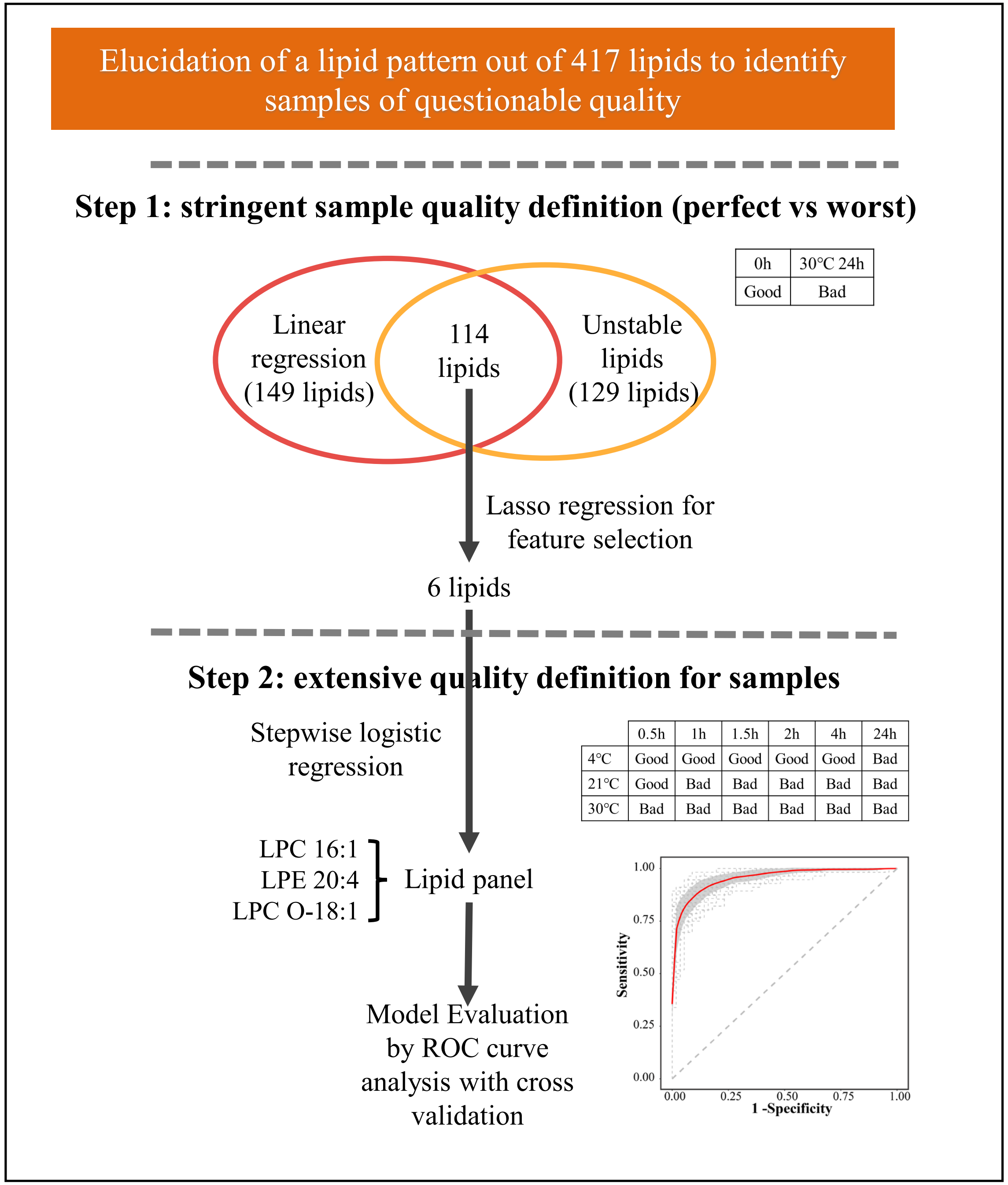

Figure S3.
Area under the receiver operating characteristic (ROC) curve (AUC) of the lipid triplet panel based on 10 times repeated 5-fold cross validation.

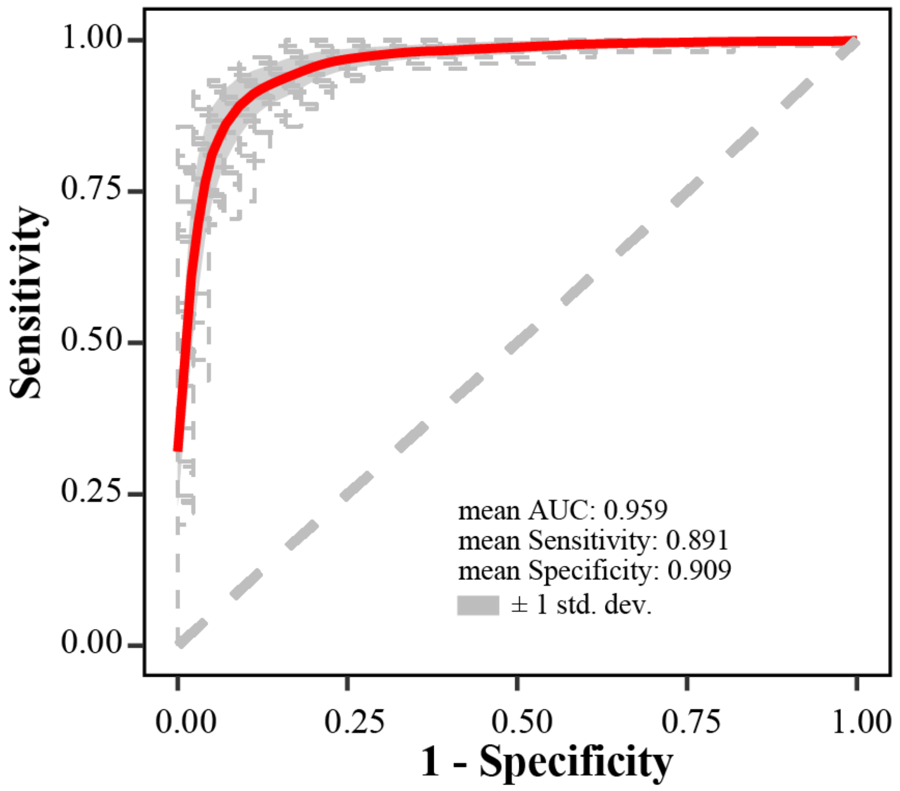

Supplement: Supplemental Figures S1–S3 [file mmc1.pdf]
