# Supplementary material for: Ex vivo instability of lipids in whole blood: preanalytical recommendations for clinical lipidomics studies
Source: J Lipid Res. 2023 Apr 21;64(6):100378. doi: 10.1016/j.jlr.2023.100378 (PMC10208886; doi:10.1016/j.jlr.2023.100378)
Supplement: Supplemental Data [file mmc3.pdf]

General Lipidomics Workflow

Overall study design

Title of the study	Ex vivo instability of lipids in whole blood – pre-analytical recommendations for clinical lipidomics studies		
Principle investigator	Guowang Xu, Rainer Lehmann		
Institution	CAS Key Laboratory of Separation Science for Analytical Chemistry, Dalian Institute of Chemical Physics, Chinese Academy of Sciences (CAS), Dalian 116023, China		
Corresponding Email	xugw@dicp.ac.cn		
Document creation date	11/30/2022	Clinical	No
Is the workflow targeted or untargeted?	Untargeted		

Lipid extraction

Extraction method	2-phase system	2-phase system	MTBE
pH adjustment	None	Were internal standards added prior extraction?	Yes

Analytical platform

Number of separation dimensions	One dimension	Ion source	ESI
Separation Type 1	LC	MS Level	MS1, MS2
Separation Mode 1	RP	Mass resolution for detected ion at MS1	High resolution
Separation window (1) for lipid analyte selection (±) in minutes	0.2	Resolution at m/z 200 at MS1	140000
RT verified by standard	No	Mass accuracy in ppm at MS1	5
CCS verified by standard	No	Mass window for precursor ion isolation (in Da total isolation window)	2
Separation of isobaric/isomeric interferece confirmed	No	Mass resolution for detected ion at MS2	High resolution
Model for separation prediction	No	Resolution at m/z 200 at MS2	70000
MS type	Orbitrap	Mass accuracy in ppm at MS2	10
MS vendor	Thermo		

Quality control

Blanks	Yes	Quality control	Yes
Type of Blanks	Injection blank	Type of QC sample	Sample pool

Method qualification and validation

Method validation	Yes	Precison	Yes
Lipid recovery	Yes	Accuracy	No
Dynamic quantification range	Yes	Guidelines followed	None
Limit of quantitation (LOQ)/Limit of detection (LOD)	Yes		

Reporting

Are reported raw data uploaded into repository?	Yes	Summary data	Quantification data
Are metadata available?	No	Raw data upload	No

Sample Descriptions

EDTA whole blood-preanalytical study (various preanalytical handling conditions for research purposes) / Human / Plasma

Provided information	Freeze-thaw cycles, Storage time (month), Time to freeze (min)	Storage time (month)	9
Temperature handling original sample	4-8 °C	Freeze-thaw cycles	0
Instant sample preparation	No	Additives	None
Time to freeze (min)	10	Were samples stored under inert gas?	No
Snap freezing in liquid N2	No	Additional preservation methods	No
Storage temperature	-80 °C	Biobank samples	No

Lipid Class Descriptions

Lipid class FA[M-H]- / Lipid identification

Lipid class	FA	MS1 verified by standard	No
MS Level	MS1	Background check at MS1	Yes
Identification level	Molecular species level	Check isomer overlap	No
Polarity mode	Negative	Lipid Identification Software	Xcalibur
Type of negative (precursor)ion	[M-H]-	Nomenclature for intact lipid molecule	Yes
Isotope correction at MS1	No		

Lipid class FA[M-H]- / For additional separation methods/analytical dimension

Quantitative	Yes	Limit of quantification	No
Internal lipid standard(s)	FA 22:0-d4	Normalization to reference	No
Type of quantification	Internal standard amount	Lipid Quantification Software	TraceFinder
Response correction	No	Batch correction	No
Type I isotope correction	No		

Lipid class DG[M+Na]+ / Lipid identification

Lipid class	DG	MS1 verified by standard	No
MS Level	MS1	Background check at MS1	Yes
Identification level	Species level	Check isomer overlap	No
Polarity mode	Positive	Lipid Identification Software	LipidSearch
Type of positive (precursor)ion	[M+Na]+	Nomenclature for intact lipid molecule	Yes
Isotope correction at MS1	No		

Lipid class DG[M+Na]+ / For additional separation methods/analytical dimension

Quantitative	Yes	Limit of quantification	No
Internal lipid standard(s)	DG (15:0/18:1)	Normalization to reference	No
Type of quantification	Internal standard amount	Lipid Quantification Software	TraceFinder
Response correction	No	Batch correction	No
Type I isotope correction	No		

Lipid class DG[M+NH4]+ / Lipid identification

Lipid class	DG	MS1 verified by standard	No
MS Level	MS1, MS2	MS2 verified by standard	No
Identification level	Species level	Background check at MS1	Yes
Polarity mode	Positive	Background check at MS2	No
Type of positive (precursor)ion	[M+NH4]+	Check isomer overlap	No
How many fragments used for ID	1 fragment	Lipid Identification Software	LipidSearch
Fragment ion 1	NL[FA-H+NH4]	Nomenclature for intact lipid molecule	Yes
Isotope correction at MS1	No	Nomenclature for fragment ions	N/A
Isotope correction at MS2	No		

Lipid class DG[M+NH4]+ / For additional separation methods/analytical dimension

Quantitative	Yes	Limit of quantification	No
Internal lipid standard(s)	DG (15:0/18:1)	Normalization to reference	No
Type of quantification	Internal standard amount	Lipid Quantification Software	TraceFinder
Response correction	No	Batch correction	No
Type I isotope correction	No		

Lipid class TG[M+Na]+ / Lipid identification

Lipid class	TG	MS1 verified by standard	No
MS Level	MS1	Background check at MS1	Yes
Identification level	Species level	Check isomer overlap	No
Polarity mode	Positive	Lipid Identification Software	LipidSearch
Type of positive (precursor)ion	[M+Na]+	Nomenclature for intact lipid molecule	Yes
Isotope correction at MS1	No		

Lipid class TG[M+Na]+ / For additional separation methods/analytical dimension

Quantitative	Yes	Limit of quantification	No
Internal lipid standard(s)	TG (15:0/15:0/15:0)	Normalization to reference	No
Type of quantification	Internal standard amount	Lipid Quantification Software	TraceFinder
Response correction	No	Batch correction	No
Type I isotope correction	No		

Lipid class TG[M+NH4]+ / Lipid identification

Lipid class	TG	Isotope correction at MS2	No
MS Level	MS1, MS2	MS1 verified by standard	No
Identification level	Species level	MS2 verified by standard	No
Polarity mode	Positive	Background check at MS1	Yes
Type of positive (precursor)ion	[M+NH4]+	Background check at MS2	No
How many fragments used for ID	3 fragments	Check isomer overlap	No
Fragment ion 1	NL[FA1-H+NH4]	Lipid Identification Software	LipidSearch
Fragment ion 2	NL[FA2-H+NH4]	Nomenclature for intact lipid molecule	Yes
Fragment ion 3	NL[FA3-H+NH4]	Nomenclature for fragment ions	N/A
Isotope correction at MS1	No		

Lipid class TG[M+NH4]+ / For additional separation methods/analytical dimension

Quantitative	Yes	Limit of quantification	No
Internal lipid standard(s)	TG (15:0/15:0/15:0)	Normalization to reference	No
Type of quantification	Internal standard amount	Lipid Quantification Software	TraceFinder
Response correction	No	Batch correction	No
Type I isotope correction	No		

Lipid class PE[M-H]- / Lipid identification

Lipid class	PE	MS1 verified by standard	No
MS Level	MS1, MS2	MS2 verified by standard	No
Identification level	Species level	Background check at MS1	Yes
Polarity mode	Negative	Background check at MS2	No
Type of negative (precursor)ion	[M-H]-	Check isomer overlap	No
How many fragments used for ID	1 fragment	Lipid Identification Software	LipidSearch
Fragment ion 1	FA-H m/z	Nomenclature for intact lipid molecule	Yes
Isotope correction at MS1	No	Nomenclature for fragment ions	N/A
Isotope correction at MS2	No		

Lipid class PE[M-H]- / For additional separation methods/analytical dimension

Quantitative	Yes	Limit of quantification	No
Internal lipid standard(s)	PE (15:0/15:0)	Normalization to reference	No
Type of quantification	Internal standard amount	Lipid Quantification Software	TraceFinder
Response correction	No	Batch correction	No
Type I isotope correction	No		

Lipid class PE[M+H]+ / Lipid identification

Lipid class	PE	MS1 verified by standard	No
MS Level	MS1, MS2	MS2 verified by standard	No
Identification level	Species level	Background check at MS1	Yes
Polarity mode	Positive	Background check at MS2	No
Type of positive (precursor)ion	[M+H]+	Check isomer overlap	No
How many fragments used for ID	1 fragment	Lipid Identification Software	LipidSearch
Fragment ion 1	NL 141.0191	Nomenclature for intact lipid molecule	Yes
Isotope correction at MS1	No	Nomenclature for fragment ions	N/A
Isotope correction at MS2	No		

Lipid class PE[M+H]+ / For additional separation methods/analytical dimension

Quantitative	No	Batch correction	No
Normalization to reference	No		

Lipid class LPC[M+H]+ / Lipid identification

Lipid class	LPC	MS1 verified by standard	No
MS Level	MS1, MS2	MS2 verified by standard	No
Identification level	Molecular species level	Background check at MS1	Yes
Polarity mode	Positive	Background check at MS2	No
Type of positive (precursor)ion	[M+H]+	Check isomer overlap	No
How many fragments used for ID	1 fragment	Lipid Identification Software	LipidSearch
Fragment ion 1	m/z 184.0733	Nomenclature for intact lipid molecule	Yes
Isotope correction at MS1	No	Nomenclature for fragment ions	N/A
Isotope correction at MS2	No		

Lipid class LPC[M+H]+ / For additional separation methods/analytical dimension

Quantitative	Yes	Limit of quantification	No
Internal lipid standard(s)	LPC 15:0; LPC 19:0	Normalization to reference	No
Type of quantification	Internal standard amount	Lipid Quantification Software	TraceFinder
Response correction	No	Batch correction	No
Type I isotope correction	No		

Lipid class LPE[M+H]+ / Lipid identification

Lipid class	LPE	MS1 verified by standard	No
MS Level	MS1, MS2	MS2 verified by standard	No
Identification level	Molecular species level	Background check at MS1	Yes
Polarity mode	Positive	Background check at MS2	No
Type of positive (precursor)ion	[M+H]+	Check isomer overlap	No
How many fragments used for ID	1 fragment	Lipid Identification Software	LipidSearch
Fragment ion 1	NL 141.0191	Nomenclature for intact lipid molecule	Yes
Isotope correction at MS1	No	Nomenclature for fragment ions	N/A
Isotope correction at MS2	No		

Lipid class LPE[M+H]+ / For additional separation methods/analytical dimension

Quantitative	Yes	Limit of quantification	No
Internal lipid standard(s)	LPC 15:0; LPC 19:0	Normalization to reference	No
Type of quantification	Internal standard amount	Lipid Quantification Software	TraceFinder
Response correction	No	Batch correction	No
Type I isotope correction	No		

Lipid class PG[M-H]- / Lipid identification

Lipid class	PG	MS1 verified by standard	No
MS Level	MS1	Background check at MS1	Yes
Identification level	Molecular species level	Check isomer overlap	No
Polarity mode	Negative	Lipid Identification Software	Xcalibur
Type of negative (precursor)ion	[M-H]-	Nomenclature for intact lipid molecule	Yes
Isotope correction at MS1	No		

Lipid class PG[M-H]- / For additional separation methods/analytical dimension

Quantitative	Yes	Limit of quantification	No
Internal lipid standard(s)	PG (15:0/15:0)	Normalization to reference	No
Type of quantification	Internal standard amount	Lipid Quantification Software	TraceFinder
Response correction	No	Batch correction	No
Type I isotope correction	No		

Lipid class Cer[M+H]+ / Lipid identification

Lipid class	Cer	MS1 verified by standard	No
MS Level	MS1, MS2	MS2 verified by standard	No
Identification level	Species level	Background check at MS1	Yes
Polarity mode	Positive	Background check at MS2	No
Type of positive (precursor)ion	[M+H]+	Check isomer overlap	No
How many fragments used for ID	1 fragment	Lipid Identification Software	LipidSearch
Fragment ion 1	Long chain base specific product ions	Nomenclature for intact lipid molecule	Yes
Isotope correction at MS1	No	Nomenclature for fragment ions	N/A
Isotope correction at MS2	No		

Lipid class Cer[M+H]+ / For additional separation methods/analytical dimension

Quantitative	Yes	Limit of quantification	No
Internal lipid standard(s)	Cer(d18:1/17:0)	Normalization to reference	No
Type of quantification	Internal standard amount	Lipid Quantification Software	TraceFinder
Response correction	No	Batch correction	No
Type I isotope correction	No		

Lipid class Cer[M+H-H2O]+ / Lipid identification

Lipid class	Cer	MS1 verified by standard	No
MS Level	MS1, MS2	MS2 verified by standard	No
Identification level	Species level	Background check at MS1	Yes
Polarity mode	Positive	Background check at MS2	No
Type of positive (precursor)ion	[M+H-H2O]+	Check isomer overlap	No
How many fragments used for ID	1 fragment	Lipid Identification Software	LipidSearch
Fragment ion 1	Long chain base specific product ions	Nomenclature for intact lipid molecule	Yes
Isotope correction at MS1	No	Nomenclature for fragment ions	N/A
Isotope correction at MS2	No		

Lipid class Cer[M+H-H2O]+ / For additional separation methods/analytical dimension

Quantitative	Yes	Limit of quantification	No
Internal lipid standard(s)	Cer(d18:1/17:0)	Normalization to reference	No
Type of quantification	Internal standard amount	Lipid Quantification Software	TraceFinder
Response correction	No	Batch correction	No
Type I isotope correction	No		

Lipid class HexCer[M+H]+ / Lipid identification

Lipid class	HexCer	MS1 verified by standard	No
MS Level	MS1, MS2	MS2 verified by standard	No
Identification level	Molecular species level	Background check at MS1	Yes
Polarity mode	Positive	Background check at MS2	No
Type of positive (precursor)ion	[M+H]+	Check isomer overlap	No
How many fragments used for ID	1 fragment	Lipid Identification Software	LipidSearch
Fragment ion 1	Long chain base specific product ions	Nomenclature for intact lipid molecule	Yes
Isotope correction at MS1	No	Nomenclature for fragment ions	N/A
Isotope correction at MS2	No		

Lipid class HexCer[M+H]+ / For additional separation methods/analytical dimension

Quantitative	Yes	Limit of quantification	No
Internal lipid standard(s)	Cer(d18:1/17:0)	Normalization to reference	No
Type of quantification	Internal standard amount	Lipid Quantification Software	TraceFinder
Response correction	No	Batch correction	No
Type I isotope correction	No		

Lipid class HexCer[M+H-H2O]+ / Lipid identification

Lipid class	HexCer	MS1 verified by standard	No
MS Level	MS1, MS2	MS2 verified by standard	No
Identification level	Molecular species level	Background check at MS1	Yes
Polarity mode	Positive	Background check at MS2	No
Type of positive (precursor)ion	[M+H-H2O]+	Check isomer overlap	No
How many fragments used for ID	1 fragment	Lipid Identification Software	LipidSearch
Fragment ion 1	Long chain base specific product ions	Nomenclature for intact lipid molecule	Yes
Isotope correction at MS1	No	Nomenclature for fragment ions	N/A
Isotope correction at MS2	No		

Lipid class HexCer[M+H-H2O]+ / For additional separation methods/analytical dimension

Quantitative	Yes	Limit of quantification	No
Internal lipid standard(s)	Cer(d18:1/17:0)	Normalization to reference	No
Type of quantification	Internal standard amount	Lipid Quantification Software	TraceFinder
Response correction	No	Batch correction	No
Type I isotope correction	No		

Lipid class SM[M+H]+ / Lipid identification

Lipid class	SM	MS1 verified by standard	No
MS Level	MS1, MS2	MS2 verified by standard	No
Identification level	Species level	Background check at MS1	Yes
Polarity mode	Positive	Background check at MS2	No
Type of positive (precursor)ion	[M+H]+	Check isomer overlap	No
How many fragments used for ID	1 fragment	Lipid Identification Software	LipidSearch
Fragment ion 1	m/z 184.0733	Nomenclature for intact lipid molecule	Yes
Isotope correction at MS1	No	Nomenclature for fragment ions	N/A
Isotope correction at MS2	No		

Lipid class SM[M+H]+ / For additional separation methods/analytical dimension

Quantitative	Yes	Limit of quantification	No
Internal lipid standard(s)	SM (d18:1/12:0)	Normalization to reference	No
Type of quantification	Internal standard amount	Lipid Quantification Software	TraceFinder
Response correction	No	Batch correction	No
Type I isotope correction	No		

Lipid class SE[M+NH4]+ / Lipid identification

Lipid class	SE	MS1 verified by standard	No
MS Level	MS1	Background check at MS1	Yes
Identification level	Molecular species level	Check isomer overlap	No
Polarity mode	Positive	Lipid Identification Software	Xcalibur
Type of positive (precursor)ion	[M+NH4]+	Nomenclature for intact lipid molecule	Yes
Isotope correction at MS1	No		

Lipid class SE[M+NH4]+ / For additional separation methods/analytical dimension

Quantitative	Yes	Limit of quantification	No
Internal lipid standard(s)	Cer (d18:1/17:0); DG (15:0/18:1); PC 30:0	Normalization to reference	No
Type of quantification	Internal standard amount	Lipid Quantification Software	TraceFinder
Response correction	No	Batch correction	No
Type I isotope correction	No		

Lipid class PC[M+H]+ / Lipid identification

Lipid class	PC	MS1 verified by standard	No
MS Level	MS1, MS2	MS2 verified by standard	No
Identification level	Species level	Background check at MS1	Yes
Polarity mode	Positive	Background check at MS2	No
Type of positive (precursor)ion	[M+H]+	Check isomer overlap	No
How many fragments used for ID	1 fragment	Lipid Identification Software	LipidSearch
Fragment ion 1	m/z 184.0733	Nomenclature for intact lipid molecule	Yes
Isotope correction at MS1	No	Nomenclature for fragment ions	N/A
Isotope correction at MS2	No		

Lipid class PC[M+H]+ / For additional separation methods/analytical dimension

Quantitative	Yes	Limit of quantification	No
Internal lipid standard(s)	PC (15:0/15:0); PC (19:0/19:0)	Normalization to reference	No
Type of quantification	Internal standard amount	Lipid Quantification Software	TraceFinder
Response correction	No	Batch correction	No
Type I isotope correction	No		

Lipid class PC[M+CH3COO]- / Lipid identification

Lipid class	PC	MS1 verified by standard	No
MS Level	MS1, MS2	MS2 verified by standard	No
Identification level	Species level	Background check at MS1	Yes
Polarity mode	Negative	Background check at MS2	No
Type of negative (precursor)ion	[M+CH3COO]-	Check isomer overlap	No
How many fragments used for ID	1 fragment	Lipid Identification Software	LipidSearch
Fragment ion 1	FA-H m/z	Nomenclature for intact lipid molecule	Yes
Isotope correction at MS1	No	Nomenclature for fragment ions	N/A
Isotope correction at MS2	No		

Lipid class PC[M+CH3COO]- / For additional separation methods/analytical dimension

Quantitative	No	Batch correction	No
Normalization to reference	No		

Lipid class PI[M-H]- / Lipid identification

Lipid class	PI	Isotope correction at MS2	No
MS Level	MS1, MS2	MS1 verified by standard	No
Identification level	Species level	MS2 verified by standard	No
Polarity mode	Negative	Background check at MS1	Yes
Type of negative (precursor)ion	[M-H]-	Background check at MS2	No
How many fragments used for ID	2 fragments	Check isomer overlap	No
Fragment ion 1	m/z 241.0122	Lipid Identification Software	LipidSearch
Fragment ion 2	FA-H m/z	Nomenclature for intact lipid molecule	Yes
Isotope correction at MS1	No	Nomenclature for fragment ions	N/A

Lipid class PI[M-H]- / For additional separation methods/analytical dimension

Quantitative	Yes	Limit of quantification	No
Internal lipid standard(s)	PG (15:0/15:0)	Normalization to reference	No
Type of quantification	Internal standard amount	Lipid Quantification Software	TraceFinder
Response correction	No	Batch correction	No
Type I isotope correction	No		
